# Association of a Comprehensive Healthy Lifestyle Score with Risk of All-Cause, Cancer, and Cardiovascular Mortality: Evidence from an 18-Year Cohort Study

**DOI:** 10.3390/nu18050856

**Published:** 2026-03-06

**Authors:** Dongmin Kim, Daeyun Kim, Hyunju Kim, Jihye Kim

**Affiliations:** 1 Department of Genetics and Biotechnology, College of Life Sciences, Kyung Hee University, Yongin 17104, Republic of Korea; kdmpre@khu.ac.kr (D.K.); daeyun5405@khu.ac.kr (D.K.); 2 Department of Epidemiology, University of Washington, Seattle, WA 98195, USA; hyunjuk1@uw.edu; 3 Cardiovascular Health Research Unit, University of Washington, Seattle, WA 98195, USA

**Keywords:** healthy lifestyle score, all-cause mortality, cancer mortality, cardiovascular disease mortality, cohort study

## Abstract

Background/Objectives: Comprehensive management of lifestyle factors is important for long-term survival. This study aims to examine whether a comprehensive healthy lifestyle score (HLS) incorporating overall diet assessment predicts all-cause, cancer, and cardiovascular mortality in Korean population. Methods: This prospective cohort study was conducted among men and women (n = 111,633, 64.6% women) aged 40 to 85 years who participated in the Korean Genome and Epidemiology Study_Health Examinees (Mean age = 55.2, SD = 8.8). Participants completed a baseline questionnaire between 2004 and 2013 and were followed until December 2023. The HLS consisted of five components classified as healthy behaviors: never or former smoking; engaging in ≥30 min/day of moderate-to-vigorous physical activity on ≥5 days/week; alcohol intake ≤40 g/day for men and ≤20 g/day for women; a BMI of 18.5–24.9 kg/m^2^; and an unhealthful plant-based diet index (uPDI) in the bottom 40th percentile, which reflects overall diet quality and aligns with the traditional plant-rich dietary pattern of Koreans. Diet was assessed using data from baseline and the first follow-up, while the remaining components were measured at baseline only. Cox proportional hazards models were applied to evaluate multivariable-adjusted associations between the HLS and all-cause, cancer, and cardiovascular mortality. Results: During 1,538,490 person-years of follow-up, 5246 all-cause deaths, 2362 cancer deaths, and 815 cardiovascular deaths were documented. Compared with the lowest HLS category, men with the highest HLS had lower risks of all-cause (HR: 0.65, 95% CI: 0.53–0.80), cancer (HR: 0.62, 95% CI: 0.46–0.85), and cardiovascular mortality (HR: 0.34, 95% CI: 0.17–0.66). Among women, the corresponding HRs were 0.38 (95% CI: 0.26–0.55), 0.52 (95% CI: 0.29–0.90), and 0.30 (95% CI: 0.11–0.84), respectively. The inverse association was stronger in older adults (≥55 years) than in younger adults. All five individual lifestyle components, including diet (quintile 5 vs. quintile 1 of uPDI: HR 0.74, 95% CI: 0.66–0.83 in men; HR 0.67, 95% CI: 0.58–0.76 in women), were significantly associated with a lower risk of all-cause mortality. However, when smoking was excluded from the HLS, the inverse association was attenuated, particularly among men. Conclusions: Greater adherence to a healthy lifestyle score was strongly associated with reduced risks of all-cause, cancer, and cardiovascular mortality. These findings underscore the importance of promoting integrated, multi-behavior lifestyle interventions, especially smoking cessation, to reduce premature mortality.

## 1. Introduction

Lifestyle behaviors are major determinants of health outcomes. In particular, modifiable lifestyle factors such as smoking, physical inactivity, diet, alcohol intake, and body mass index (BMI) are associated with lower risk of chronic diseases including cardiovascular disease, cancer, and type 2 diabetes [1]. These chronic diseases account for a significant portion of mortality worldwide [2]. Additionally, following a healthy lifestyle is linked to longer life expectancy [3]. A substantial body of epidemiologic evidence has shown that individual lifestyle behaviors are strongly associated with all-cause and cause-specific mortality [2,4,5,6,7].

Recently, research on individual lifestyle factors has been enhanced by a more comprehensive approach, utilizing a composite score or healthy lifestyle score (HLS) to integrate lifestyle factors and assess adherence to a healthy lifestyle [8,9,10]. A systematic review and meta-analysis showed that adherence to a healthy lifestyle score including diet, alcohol, BMI, and physical activity was associated with lower risk of all-cause mortality [11]. Another meta-analysis found that adherence to a healthy lifestyle score composed of four or five lifestyle factors was associated with lower risk of cancer mortality [12]. However, most studies addressing combined lifestyle factors and mortality have been carried out in Western populations and evidence is limited in Asian populations, who have differences in lifestyle patterns, genetic backgrounds, and disease profiles [2,13,14].

Furthermore, prior studies have lacked comprehensive dietary assessments or relied on limited food items that do not reflect overall diet quality in Asian countries [12]. A city-wide prospective study conducted in China reported inverse associations between a healthy lifestyle score and the risk of all-cause mortality; however, the assessment did not include dietary factors [15]. The Japan Collaborative Cohort Study incorporated only a limited number of food items, such as fruit, fish, milk, vegetables, and beans, for dietary evaluation [16]. Given that diet is a major modifiable determinant of chronic diseases and mortality [17,18], lifestyle indices incorporating validated dietary assessments can provide more accurate and culturally relevant evidence. In this study, we applied a plant-based diet index to evaluate overall diet quality. Plant-based diet indices, such as the healthful plant-based diet index (hPDI) and the unhealthful plant-based diet index (uPDI), assess overall diet quality by distinguishing the healthfulness of plant-derived foods. Adherence to the hPDI, which assigns positive scores to healthy plant foods and negative scores to unhealthy plant foods and animal-derived foods, has been associated with a lower risk of mortality. In contrast, adherence to the uPDI, which inversely scores plant-based food categories, has been linked to a higher risk of mortality in large prospective cohorts, including Korean populations [18,19,20].

Although traditional Korean diets are predominantly plant-based, certain plant foods contribute to high sodium intake due to the frequent consumption of preserved vegetables. The plant-based diet index used in this study therefore enables differentiation of food groups according to the quality of plant-derived foods.

In this context, we constructed a comprehensive HLS incorporating smoking status, physical activity, alcohol consumption, BMI, and overall diet quality index based on plant-based diet index and evaluated the association between adherence to healthy lifestyle behaviors and the risks of all-cause, cancer, and cardiovascular mortality using data from a large, population-based prospective cohort of Korean adults with linkage to national death records.

## 2. Materials and Methods

### 2.1. Study Population

The data were obtained from the Korean Genome and Epidemiology Study_Health Examinees (Koges_HEXA), a population-based cohort study designed to investigate non-communicable chronic diseases and lifestyle-related risk factors among Korean adults [21]. The cohort recruited 173,195 individuals aged 40–85 years from major metropolitan and mid-sized cities across Korea. Baseline examinations were conducted between 2004 and 2013 and mortality information was updated through December 2023. All participants provided written informed consent, and the study protocol was approved by the Institutional Review Board of Kyung Hee University (KHGIRB-25-278(RA)).

Participants with cardiovascular disease (myocardial infarction, stroke, or angina) or any cancer at baseline were excluded (n = 11,191). Participants without linked mortality data were excluded (n = 39,899). We also excluded individuals without information on any HLS components (n = 5438). Those reporting implausible total energy intake (<800 or >4200 kcal/day for men, and <500 or >3500 kcal/day for women) were also excluded (n = 2091). Individuals with missing covariate information were removed (n = 2943). Finally, a total of 111,633 participants were included in the final analysis (Supplementary Figure S1).

### 2.2. Ascertaining a Healthy Lifestyle Score

Lifestyle information was collected at baseline through a standardized self-administered questionnaire. Using these data, we developed an HLS by allocating 1 point to each of five lifestyle behaviors when the participant met the predefined “healthy” criterion, and 0 otherwise. The five components included smoking status, physical activity, BMI, alcohol intake, and dietary quality. Participants who were never smokers or former smokers were categorized as meeting the healthy smoking criterion. Individuals who performed ≥30 min/day of moderate-to-vigorous activity on at least 5 days/week were classified as physically active [22]. For alcohol consumption, we defined a healthy pattern as being a never or former drinker, or a current drinker with consumption ≤40 g/day for men and ≤20 g/day for women [23,24,25]. BMI was obtained from measured height and weight, and values within 18.5–24.9 kg/m^2^ were classified as healthy [26]. These four lifestyle factors were measured at baseline only, but dietary intake was assessed using a validated food frequency questionnaire administered at baseline (2004–2013) and the first follow-up examination, which was carried out between 2012 and 2016. To assess overall diet quality, we used the uPDI, which showed a stronger association with mortality than the hPDI in KoGES_HEXA [18]. FFQ items were grouped into 17 food groups and classified as healthy plant (whole grain, fruits, vegetables, nuts, legumes, tea and coffee), less healthy plant (potatoes, sugar-sweetened beverages, sweets and desserts, salty foods), or animal foods (animal fat, dairy, eggs, fish or seafood, meat, miscellaneous animal foods). Each food group was assigned a score from 1 to 5 based on intake quintiles. For the uPDI, healthy plant foods were reverse-scored (5–1), and less healthy plant and animal foods were positively scored (1–5). Scores were summed to obtain a total uPDI ranging from 17 to 85 [27]. For the HLS dietary component, individuals in the bottom 40th percentile were classified as having a healthy diet; in secondary analyses, those in the lowest 25th percentile were used.

By summing the score from five lifestyle components, total scores ranged from 0 to 5, with higher values reflecting better adherence to overall healthy lifestyle patterns [9,22]. Additionally, a weighted HLS (range: 0–20 points) was developed by assigning graded scores to each component based on the degree of adherence: smoking (never = 4, former = 2, current = 0), alcohol consumption (none = 4, light = 3, moderate = 2, heavy = 0), physical activity (active [≥5 times/week and ≥30 min/session] = 4, moderately active [≥5 times/week and <30 min/session, or 2–4 times/week and ≥30 min/session] = 3, moderately inactive [1–2 times/week and <30 min/session] = 1, no exercise = 0), BMI (18.5–22.9 kg/m^2^ = 4, 23–24.9 kg/m^2^ = 3, 25–29.9 kg/m^2^ = 2, ≥30 kg/m^2^ = 1, <18.5 kg/m^2^ = 0), and diet quality (uPDI quintiles: quintile 1 = 4 to quintile 5 = 0).

### 2.3. Ascertainment of Death

Mortality information was obtained through the death certificate database provided by Ministry of Data and Statistics from baseline to December 2023. Causes of death were identified and classified according to the International Classification of Diseases, 10th Revision (ICD-10). All-cause mortality was defined as death from any cause recorded in the national death registry, and cardiovascular disease mortality with ICD codes I05-I79 and cancer mortality with codes C00-C97 [28].

### 2.4. Covariates

Participants at baseline completed structured questionnaires to report demographic characteristics (age and education), history of diseases (hypertension and dyslipidemia), family history (cardiovascular disease and cancer), menopausal status, and hormone replacement therapy use. History of diseases was evaluated by self-reported physician’s diagnosis and the presence of baseline diabetes included physician’s diagnosis of type 2 diabetes, fasting glucose level ≥126 mg/dL, or use of glucose-lowering medications or insulin treatment.

### 2.5. Statistical Analysis

Baseline characteristics were summarized as means and standard deviations for continuous variables and as frequencies and percentages for categorical variables. For continuous variables, the Kolmogorov–Smirnov test was used based on the normality of the variable distribution. Person-years of follow-up were calculated from baseline to the date of death or the end of follow-up, whichever occurred first.

Multivariable Cox proportional hazards regression models were used to estimate hazard ratios (HRs) and 95% confidence intervals (CIs) for the association between the HLS and all-cause mortality. All analyses were conducted separately for men and women. Models were adjusted for age (years), educational level (<7, 7–12, or >12 years), history of hypertension (yes/no), history of hyperlipidemia (yes/no), family history of cardiovascular disease (yes/no), family history of cancer (yes/no), baseline diabetes (yes/no), and menopausal status (yes/no) and hormone replacement therapy use (yes/no) for women only. These variables were selected based on prior literature and their well-established associations with mortality risk [29,30,31]. The proportional hazards assumption was evaluated using Schoenfeld residuals, and no violations were detected. P value for trend was calculated using HLS as an ordinal variable.

To examine potential effect modification, stratified analyses were performed across key demographic and clinical subgroups. Participants were stratified by age (<55 vs. ≥55 years), family history of cancer (yes/no), family history of cardiovascular disease (yes/no), history of hypertension (yes/no), history of hyperlipidemia (yes/no), and baseline diabetes status (yes/no). Additionally, we examined whether a specific HLS component was associated with mortality outcomes. Sensitivity analyses were performed to assess the robustness of the primary findings under alternative HLS definitions. First, given the observation that overweight men exhibited lower all-cause mortality compared with normal-weight men, a modified HLS was constructed in which overweight (BMI 25–29.9 kg/m^2^) was additionally scored as healthy for the BMI component. Second, a modified HLS was constructed using the lowest quartile (instead of the bottom 40th percentile) of the uPDI as the diet criterion. Third, a weighted HLS (range: 0–20 points) was developed by assigning graded scores to each component based on the degree of adherence. To minimize the potential influence of reverse causation, we repeated primary analyses after excluding deaths occurring within the first 2 years of follow-up and again after excluding deaths within the first 4 years of follow-up. All analyses were performed using SAS version 9.4 (SAS Institute, Cary, NC, USA), with two-sided *p* values < 0.05 considered statistically significant.

## 3. Results

Baseline characteristics of the study population across categories of the HLS are presented in Table 1. Individuals with higher HLS tended to be older, more highly educated, and to exhibit more favorable lifestyle profiles, including lower prevalence of smoking and heavy alcohol consumption in both men and women. The baseline characteristics of the overall cohort and the analytic cohort, stratified by sex, are presented (Supplementary Table S1).

During 1,538,490 person-years of follow-up, 5246 all-cause deaths, 2362 cancer deaths, and 815 cardiovascular deaths were documented. Higher adherence to healthy lifestyle behaviors was consistently associated with lower mortality risk. In the multivariable-adjusted model, among men, participants in the highest HLS category (score 5) had a 35% lower all-cause mortality risk compared with those in the lowest category (HR: 0.65, 95% CI: 0.53–0.80; P for trend < 0.001). Among women, the corresponding reduction was 62% (HR: 0.38, 95% CI: 0.26–0.55; P for trend < 0.001) (Table 2).

Similar inverse associations were observed for cause-specific mortality. Compared with the least healthy group, men with an HLS of 5 had a 38% lower cancer mortality risk (HR: 0.62, 95% CI: 0.46–0.85; P for trend < 0.001), and women had a 48% lower risk (HR: 0.52, 95% CI: 0.29–0.90; P for trend = 0.002). For cardiovascular mortality, the highest HLS category showed a 66% lower risk in men (HR: 0.34, 95% CI: 0.17–0.66; P for trend = 0.003) and a 70% lower risk in women (HR: 0.30, 95% CI: 0.11–0.84; P for trend = 0.011). The dose–response trend across HLS categories was consistent for both cancer and cardiovascular deaths in both sexes (Table 2). To further confirm the robustness of these associations, we additionally performed an analysis excluding participants with baseline diabetes (Supplementary Table S2). The results were generally comparable to those of the primary analysis.

**Figure 1:**
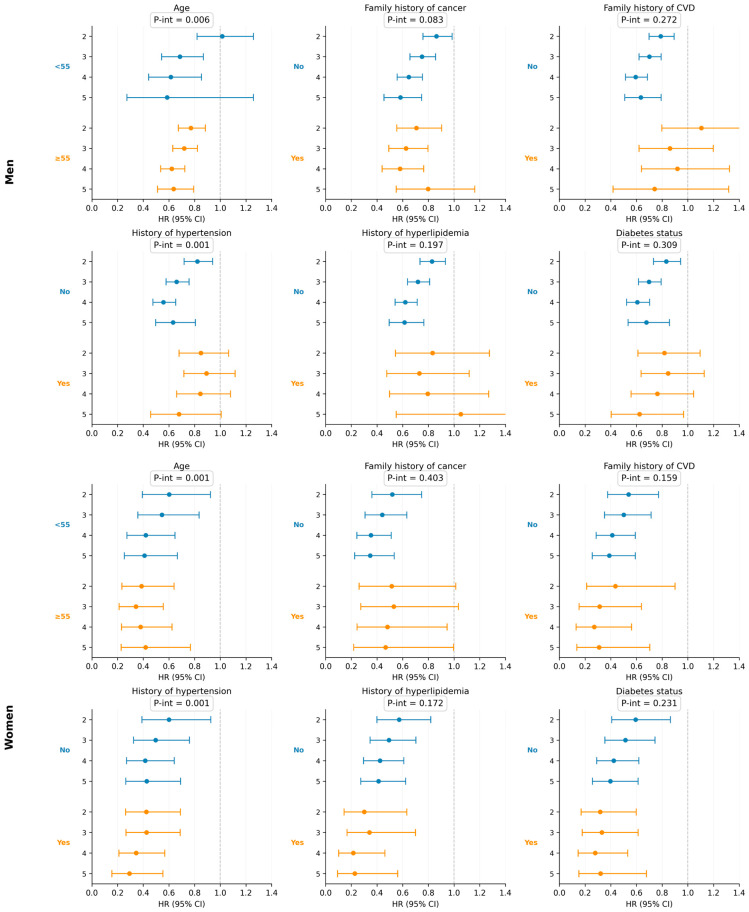
Association between healthy lifestyle scores and all-cause mortality stratified by potential risk factors. Models were adjusted for age (years), educational level (<7 years, 7–12 years, >12 years), history of hypertension (yes/no), history of hyperlipidemia (yes/no), family history of cardiovascular disease (yes/no), family history of cancer (yes/no), baseline diabetes (yes/no), and menopausal status (yes/no) and hormone replacement therapy use (yes/no) for women only.

Stratified analyses by potential risk factors demonstrated broadly consistent associations across subgroups, except for age and history of hypertension (Figure 1). The inverse association between HLS and all-cause mortality appeared stronger in older adult (≥55 years) than in younger adults (<55 years) in both sexes (P interaction = 0.006 for men and 0.001 for women). Additionally, effect modification by history of hypertension was observed, although the patterns differed by sex. In addition to the overall lifestyle score, each individual lifestyle component was also evaluated (Table 3). Leave-one-out analyses yielded generally consistent results (Table 4). When physical activity, BMI, alcohol, or diet was excluded from the lifestyle score, the inverse association with all-cause mortality remained statistically significant in both sexes. However, when smoking was excluded, the inverse association was attenuated in men (P for trend = 0.224), whereas it remained significant in women (P for trend < 0.001).

Sensitivity analyses supported the robustness of these findings. Using a modified HLS that additionally classified overweight as healthy for BMI (Supplementary Table S3), a modified HLS based on the lowest quartile of the uPDI (Supplementary Table S4), and a weighted HLS (0–20 points) (Supplementary Table S5) yielded consistent results. Furthermore, excluding deaths that occurred within the first 2 years, or 4 years of follow-up did not materially alter the associations between HLS and mortality outcomes (Supplementary Table S6).

## 4. Discussion

In this large prospective cohort study of Korean adults, we observed a clear inverse association between adherence to a healthy lifestyle and the risk of all-cause, cancer, and cardiovascular mortality in both men and women. Among men, the highest HLS category was associated with 35%, 38%, and 66% lower risks of all-cause, cancer, and cardiovascular mortality, respectively after adjustment for a broad range of sociodemographic and clinical factors. Among women, the corresponding reductions were 62%, 48%, and 70%. The magnitude of the associations was stronger in older adult (≥55 years) than in younger adults (<55 years) in both sexes. Individual lifestyle factors were significantly associated with risk of all-cause mortality including diet. However, when smoking was removed from the HLS, the inverse association was attenuated, particularly among men. Together, these findings suggest that healthier lifestyle profiles confer substantial survival benefits across diverse subpopulations, with a particularly strong emphasis on the importance of smoking cessation.

Our findings are consistent with previous cohort studies demonstrating that holistic improvement in lifestyle behaviors can substantially reduce mortality risk. In the U.S. Nurses’ Health Study and Health Professionals Follow-up Study, adherence to five low-risk lifestyle factors was associated with a 74% lower risk of all-cause mortality, as well as 82% and 65% reductions in cardiovascular and cancer mortality, respectively, over up to 34 years of follow-up [30]. Similarly, a review of the European Prospective Investigation into Cancer and Nutrition cohorts reported that adherence to four healthy lifestyle behaviors—diet, alcohol consumption, BMI, and physical activity—was consistently associated with lower cancer mortality [8]. These inverse associations have also been observed among individuals with type 2 diabetes; in the UK Biobank, higher adherence to a healthy lifestyle score was associated with 58%, 65%, and 43% lower all-cause, cancer, and cardiovascular mortality, respectively [29]. By integrating multiple lifestyle components—including diet quality, physical activity, smoking status, alcohol use, and BMI—into a single lifestyle index, our study extends prior evidence by demonstrating substantial reductions in mortality risk in an Asian population with distinct lifestyle patterns and disease epidemiology.

The biological mechanisms underlying the observed associations are supported by previous research on individual lifestyle factors and mortality. Smoking is a well-established driver of oxidative stress, systemic inflammation, endothelial dysfunction, and DNA damage, collectively increasing mortality risk from multiple causes [32,33]. Regular physical activity improves vascular structure and function, enhances endothelial responsiveness, and reduces insulin resistance, thereby lowering the risks of cancer and cardiovascular mortality [34,35]. Maintaining a healthier BMI is associated with lower visceral adiposity and reduced chronic inflammation, both of which are strongly linked to mortality from cardiometabolic diseases [36]. Excessive alcohol consumption induces hepatic and pancreatic injury, disrupts metabolic homeostasis, and elevates the risk of cancer and cardiovascular events [37,38]. Finally, diet quality plays a key role in modulating metabolic pathways relevant to long-term survival. Diets rich in healthy plant-based foods—such as whole grains, legumes, fruits, vegetables, and nuts—are known to reduce systemic inflammation, improve lipid profiles, and decrease oxidative stress, thereby contributing to lower risks of cardiovascular disease and cancer [18,39].

These lifestyle components influence mortality through overlapping yet distinct biological pathways. Thus, individuals who adhere to multiple healthy lifestyle behaviors—including nonsmoking, regular physical activity, healthy adiposity, moderate alcohol use, and high-quality diet—may experience cumulative benefits in reducing the incidence of metabolic diseases and associated mortality. Accordingly, this study suggests that improving lifestyle habits in an integrated manner, particularly under the condition of smoking cessation, can help reduce mortality rates, rather than focusing on single behavior. 

The association between the HLS and all-cause mortality was stronger in women than in men. Women may be more biologically sensitive to lifestyle-related risk factors. In a population-based cohort of US adults, women compared to men derived greater gains in all-cause and cardiovascular mortality risk reduction from equivalent doses of regular exercise [40]. Association between healthy dietary pattern and all-cause mortality was stronger in women than men as well [41]. Women generally have lower baseline mortality rates [42] and adopt healthier behaviors (e.g., less smoking and healthier diet) more consistently and accurately, making the lifestyle score a more precise reflection of their true long-term exposure. In addition, hormonal factors, body composition, and metabolic differences may also modify how lifestyle factors influence disease risk, leading to stronger protective effects in women [43].

Given the one-component-out analysis in this study, smoking was a major contributor to the HLS–mortality association among Korean men. The smoking rate is substantially higher in men than in women in Korea (28.5% vs. 4.2%) [44]. Cigarette smokers are at a higher risk of all-cause and cancer mortality compared with non-smokers [45]. Therefore, smoking may attenuate the benefits of other healthy lifestyle habits on mortality, particularly among men.

The inverse association between HLS and all-cause mortality was more evident in older adults than in younger adults. Healthier lifestyle habits in older individuals such as lower smoking prevalence and more nutritious dietary patterns, may therefore exert a stronger influence on mortality risk in this population. Smoking rates have been rising among younger adults in Korea, with the highest prevalence reported in those in their 40s [44]. Additionally, data from the Korea National Health and Nutrition Examination Survey indicate that adults aged ≥50 years maintain higher diet quality than adults aged 40–49 years, as reflected in their Healthy Eating Index scores.

Interestingly, overweight men exhibited a lower risk of all-cause mortality compared with normal weight men. This phenomenon, often referred to as the “obesity paradox,” has been documented in overweight or obese individuals with established chronic conditions, particularly cardiovascular and kidney diseases, as well as in older adults [46]. Nevertheless, these findings should be interpreted cautiously, as obesity remains a major risk factor for the development of cardiometabolic disorders. It is also plausible that overweight individuals may engage in more favorable health-related behaviors such as adhering to a healthier diet, quitting smoking, or increasing physical activity, because they may be more attentive to their overall health status.

The strengths of this study include the use of a large population-based cohort, a longer follow up period (≈18 years), and validated and repeated dietary assessments. Particularly, the incorporation of an overall diet quality index and detailed comprehensive assessment of multiple lifestyle factors further enhances the robustness of our findings. In addition, the consistency of associations across subgroups and sensitivity analyses supports the reliability of the estimated effects. However, several limitations warrant consideration. First, lifestyle factors were measured at baseline only, and potential changes during follow-up might not be captured. Second, the use of self-reported questionnaires for some lifestyle factors may have measurement errors. Third, potential misclassification could have occurred, such as former drinkers being grouped with never drinkers in the healthy category. Fourth, approximately one-third of participants were excluded from the analytic sample, which may have influenced the observed associations. Finally, although we adjusted for a comprehensive set of covariates, residual confounders such as income or healthcare access might not be entirely considered.

## 5. Conclusions

In this large, population-based cohort of Korean adults, greater adherence to a healthy lifestyle was strongly associated with lower risks of all-cause, cancer, and cardiovascular mortality. These findings underscore the importance of adopting and maintaining a comprehensive lifestyle approach, rather than targeting single behaviors in isolation, with particular emphasis on smoking cessation and adherence to a healthy plant-based diet for reducing mortality risk. Future studies are warranted to elucidate the biological mechanisms underlying the associations between multidimensional lifestyle behaviors and long-term survival, and to further explore sex-specific differences in these biological responses.

## Figures and Tables

**Table 1:** Baseline characteristics of participants by healthy lifestyle score category.

Healthy Lifestyle Score						
Men	0–1	2	3	4	5	*p*-value
Participants	5158 (13.08)	12,243 (31.06)	13,942 (35.37)	6725 (17.06)	1355 (3.44)	
Age (years)	52.39 (8.57)	54.28 (9.16)	56.30 (9.34)	58.76 (9.05)	60.98 (8.88)	<0.001
Educational level, years						<0.001
<7	529 (10.26)	1179 (9.63)	1152 (8.26)	441 (6.56)	60 (4.43)	
7–12	2546 (49.36)	5707 (46.61)	6176 (44.30)	2836 (42.17)	547 (40.37)	
>12	2083 (40.38)	5357 (43.76)	6614 (47.44)	3448 (51.27)	748 (55.20)	
Smoking						<0.001
Never	224 (4.34)	2516 (20.55)	4871 (34.94)	2670 (39.70)	532 (39.26)	
Former	563 (10.92)	3924 (32.05)	6672 (47.86)	3723 (55.36)	823 (60.74)	
Current	4371 (84.74)	5803 (47.40)	2399 (17.21)	332 (4.94)	0	
Physical activity, (min/week)						<0.001
<150	5074 (98.37)	11,602 (94.76)	11,878 (85.20)	3680 (54.72)	0	
≥150	84 (1.63)	641 (5.24)	2064 (14.80)	3045 (45.28)	1355 (100)	
Alcohol consumption (g/d)	45.98 (70.87)	20.90 (38.01)	12.33 (22.41)	9.60 (16.77)	7.66 (10.42)	<0.001
BMI (kg/m^2^)	25.99 (2.89)	25.00 (2.91)	23.90 (2.48)	23.40 (2.03)	22.93 (1.47)	<0.001
uPDI	56.75 (5.33)	54.78 (6.28)	52.10 (6.40)	48.90 (5.10)	47.13 (4.17)	<0.001
History of hypertension	1159 (22.47)	2590 (21.15)	2814 (20.18)	1509 (22.44)	315 (23.25)	0.001
History of hyperlipidemia	535 (10.37)	1218 (9.95)	1278 (9.17)	639 (9.50)	120 (8.86)	0.056
T2D incidence	424 (8.22)	946 (7.73)	1116 (8.00)	685 (10.19)	199 (14.69)	<0.001
Women	0–1	2	3	4	5	*p*-value
Participants	723 (1.00)	13,000 (18.00)	34,000 (47.08)	20,538 (28.44)	3949 (5.47)	
Age (years)	52.08 (7.79)	55.81 (8.84)	54.49 (8.57)	54.62 (8.14)	55.87 (7.90)	<0.001
Educational level, years						<0.001
<7	178 (24.62)	3941 (30.24)	6442 (18.95)	2279 (11.10)	389 (9.85)	
7–12	465 (64.32)	6934 (53.34)	19,001 (55.89)	11,760 (57.26)	2413 (61.10)	
>12	80 (11.07)	2135 (16.42)	8557 (25.17)	6499 (31.64)	1147 (29.05)	
Smoking						<0.001
Never	240 (33.20)	12,044 (92.65)	33,152 (97.51)	20,233 (98.51)	3899 (98.73)	
Former	17 (2.35)	178 (1.37)	423 (1.24)	247 (1.20)	50 (1.27)	
Current	466 (64.45)	778 (5.98)	425 (1.25)	58 (0.28)	0	
Physical activity, (min/week)						<0.001
<150	713 (98.62)	12,912 (99.32)	31,624 (93.01)	14,288 (69.57)	0	
≥150	10 (1.38)	88 (0.68)	2376 (6.99)	6250 (30.43)	3949 (100)	
Alcohol consumption (g/d)	27.77 (52.44)	3.24 (13.07)	1.61 (6.39)	1.33 (4.16)	1.21 (3.17)	<0.001
BMI (kg/m^2^)	26.12 (3.92)	26.20 (3.39)	23.24 (2.70)	22.61 (2.04)	22.33 (1.56)	<0.001
uPDI	46.94 (4.80)	47.81 (4.45)	49.73 (5.83)	56.11 (4.46)	58.52 (3.76)	<0.001
History of hypertension	166 (22.96)	3250 (25.00)	5269 (15.50)	2763 (13.45)	556 (14.08)	<0.001
History of hyperlipidemia	75 (10.37)	1374 (10.57)	2909 (8.56)	1820 (8.86)	422 (10.69)	<0.001
Menopause status	324 (44.81)	4318 (33.22)	13,612 (40.04)	8541 (41.59)	1374 (34.79)	<0.001
HRT use	81 (11.20)	1750 (13.46)	4855 (14.28)	3254 (15.84)	759 (19.22)	<0.001
T2D incidence	48 (6.64)	846 (6.51)	1504 (4.42)	936 (4.56)	235 (5.95)	<0.001

HRT: Hormone replacement therapy.

**Table 2:** Association between healthy lifestyle scores and the risk of all-cause and cause-specific mortality.

	Men		Women		
	No. of Cases/Total	HR (95% CI)	No. of Cases/Total	HR (95% CI)	P Interaction
All-cause mortality	3063/39,423		2183/72,210		
0–1	416	1 (ref)	40	1 (ref)	0.094
2	955	0.83 (0.74–0.93)	551	0.52 (0.38–0.72)	
3	1057	0.72 (0.64–0.81)	1032	0.46 (0.34–0.63)	
4	513	0.63 (0.55–0.72)	468	0.38 (0.28–0.53)	
5	122	0.65 (0.53–0.80)	92	0.38 (0.26–0.55)	
P for trend	<0.001		<0.001		
1 point increment	0.87 (0.84–0.91)		0.85 (0.81–0.90)		
Cancer mortality	1319/39,423		1043/72,210		
0–1	187	1 (ref)	16	1 (ref)	0.811
2	421	0.81 (0.68–0.96)	244	0.63 (0.38–1.04)	
3	440	0.65 (0.55–0.78)	482	0.54 (0.33–0.89)	
4	217	0.58 (0.48–0.71)	247	0.48 (0.29–0.79)	
5	54	0.62 (0.46–0.85)	54	0.52 (0.29–0.90)	
P for trend	<0.001		0.002		
1 point increment	0.85 (0.81–0.90)		0.89 (0.82–0.96)		
Cardiovascular disease mortality	457/39,423		358/72,210		
0–1	65	1 (ref)	6	1 (ref)	0.888
2	138	0.76 (0.56–1.02)	101	0.55 (0.24–1.25)	
3	168	0.73 (0.54–0.97)	169	0.49 (0.21–1.10)	
4	76	0.59 (0.42–0.83)	72	0.42 (0.18–0.96)	
5	10	0.34 (0.17–0.66)	10	0.30 (0.11–0.84)	
P for trend	0.003		0.011		
1 point increment	0.84 (0.76–0.92)		0.84 (0.74–0.96)		

Models were adjusted for age (years), educational level (<7 years, 7–12 years, >12 years), history of hypertension (yes/no), history of hyperlipidemia (yes/no), family history of cardiovascular disease (yes/no), family history of cancer (yes/no), baseline diabetes (yes/no), and menopausal status (yes/no) and hormone replacement therapy use (yes/no) for women only.

**Table 3:** Association between individual healthy lifestyle components and risk for all-cause mortality.

	Men		Women	
Category	No. of Cases/Total	HR (95% CI)	No. of Cases/Total	HR (95% CI)
Smoking				
Current	1151/12,905	Ref	89/1727	Ref
Previous	1179/15,705	0.60 (0.56–0.66)	35/915	0.67 (0.45–0.99)
Never	733/10,813	0.53 (0.48–0.58)	2059/69,568	0.44 (0.35–0.54)
Physical activity				
Irregular	1935/24,450	Ref	1554/47,819	Ref
Regular	1128/14,973	0.85 (0.78–0.91)	629/24,391	0.78 (0.71–0.85)
BMI				
BMI < 18.5	103/507	2.48 (2.03–3.02)	57/1569	1.85 (1.42–2.41)
18.5 ≤ BMI < 25	1895/23,211	Ref	1338/50,479	Ref
25 ≤ BMI < 30	996/14,642	0.84 (0.78–0.91)	690/18,142	1.02 (0.92–1.12)
BMI ≥ 30	69/1063	0.97 (0.76–1.24)	98/2020	1.21 (0.98–1.49)
Alcohol consumption *				
Heavy	454/5825	Ref	41/1695	Ref
Moderate	642/8841	0.95 (0.84–1.07)	34/1915	0.71 (0.45–1.11)
Light	975/14,361	0.84 (0.75–0.94)	383/18,729	0.76 (0.55–1.04)
Never	991/10,386	0.96 (0.86–1.07)	1725/49,867	0.85 (0.63–1.17)
Diet (uPDI)				
Quintile 5	752/8210	Ref	648/13,990	Ref
Quintile 4	636/7921	0.89 (0.80–0.99)	439/14,426	0.75 (0.67–0.85)
Quintile 3	514/7098	0.80 (0.71–0.89)	362/13,048	0.73 (0.64–0.84)
Quintile 2	635/8518	0.81 (0.73–0.90)	407/16,147	0.71 (0.62–0.80)
Quintile 1	526/7676	0.74 (0.66–0.83)	327/14,599	0.67 (0.58–0.76)

Models were adjusted for age (years), educational level (<7 years, 7–12 years, >12 years), history of hypertension (yes/no), history of hyperlipidemia (yes/no), family history of cardiovascular disease (yes/no), family history of cancer (yes/no), baseline diabetes (yes/no), and menopausal status (yes/no) and hormone replacement therapy use (yes/no) for women only. * Alcohol consumption categories were defined as follows: never (0 g/day), light (men: 0 < alcohol ≤ 15 g/day; women: 0 < alcohol ≤ 10 g/day), moderate (men: 15 < alcohol ≤ 40 g/day; women: 10 < alcohol ≤ 20 g/day), and heavy (men: alcohol > 40 g/day; women: alcohol > 20 g/day).

**Table 4:** Association between healthy lifestyle score and risk of all-cause mortality after leaving out one lifestyle factor.

		No. of Cases/Total	0	1	2	3	4	P for Trend	Per 1-Point Increase
Leave-one-out									
Smoking	Men	3063/39,423	ref	0.96 (0.78–1.18)	1.03 (0.84–1.26)	0.88 (0.72–1.09)	0.98 (0.76–1.25)	0.224	0.98 (0.94–1.02)
	Women	2183/72,210	ref	0.91 (0.47–1.76)	0.82 (0.43–1.59)	0.68 (0.35–1.32)	0.68 (0.34–1.34)	<0.001	0.88 (0.83–0.93)
Exercise	Men	3063/39,423	ref	0.98 (0.75–1.29)	0.83 (0.64–1.08)	0.71 (0.54–0.92)	0.58 (0.44–0.76)	<0.001	0.85 (0.82–0.88)
	Women	2183/72,210	ref	0.88 (0.27–2.82)	0.48 (0.15–1.49)	0.41 (0.13–1.27)	0.36 (0.12–1.12)	<0.001	0.84 (0.79–0.89)
BMI	Men	3063/39,423	ref	0.83 (0.70–0.99)	0.59 (0.50–0.69)	0.52 (0.44–0.62)	0.51 (0.41–0.63)	<0.001	0.82 (0.79–0.85)
	Women	2183/72,210	ref	1.26 (0.51–3.12)	0.66 (0.28–1.60)	0.55 (0.23–1.32)	0.50 (0.21–1.22)	<0.001	0.81 (0.76–0.86)
Alcohol	Men	3063/39,423	ref	0.83 (0.72–0.95)	0.70 (0.61–0.81)	0.61 (0.52–0.71)	0.61 (0.49–0.76)	<0.001	0.87 (0.83–0.90)
	Women	2183/72,210	ref	0.48 (0.31–0.73)	0.42 (0.27–0.63)	0.35 (0.23–0.53)	0.33 (0.21–0.53)	<0.001	0.85 (0.81–0.90)
Diet	Men	3063/39,423	ref	0.95 (0.75–1.21)	0.74 (0.59–0.93)	0.66 (0.52–0.83)	0.67 (0.52–0.86)	<0.001	0.88 (0.84–0.91)
	Women	2183/72,210	ref	0.53 (0.21–1.34)	0.32 (0.13–0.78)	0.30 (0.12–0.72)	0.25 (0.10–0.61)	<0.001	0.86 (0.81–0.92)

Models were adjusted for age (years), educational level (<7 years, 7–12 years, >12 years), history of hypertension (yes/no), history of hyperlipidemia (yes/no), family history of cardiovascular disease (yes/no), family history of cancer (yes/no), baseline diabetes (yes/no), and menopausal status (yes/no) and hormone replacement therapy use (yes/no) for women only.

## Data Availability

Data underlying the results of our study are not publicly available due to KoGES data policy. Data are available from the Division of Genetic Epidemiology and Health Index, NIH, Korea Disease Control and Prevention Agency (contact via So Ra Kwon at sora9360@korea.kr) for researchers who meet the criteria for access to confidential data.

## Supplementary Materials

The following supporting information can be downloaded at: https://www.mdpi.com/article/10.3390/nu18050856/s1, Figure S1: Flow chart of inclusion and exclusion of the study population. Table S1: Baseline characteristics for the overall and included cohorts by sex. Table S2: Association between healthy lifestyle scores and the risk of mortality after excluding participants with baseline diabetes. Table S3: Association between a BMI category-modified healthy lifestyle score and the risk of all-cause and cause-specific mortality. Table S4: Association between a diet cutoff-modified healthy lifestyle score (25th percentile of uPDI) and the risk of all-cause and cause-specific mortality. Table S5: Association between weighted healthy lifestyle score (0–20 points) and the risk of all-cause and cause-specific mortality. Table S6: 2-year or 4-year lag analysis of the association between healthy lifestyle score and all-cause mortality risk.
