# Supplementary material for: Association of a Comprehensive Healthy Lifestyle Score with Risk of All-Cause, Cancer, and Cardiovascular Mortality: Evidence from an 18-Year Cohort Study
Source: Nutrients. 2026 Mar 6;18(5):856. doi: 10.3390/nu18050856 (PMC12986665; doi:10.3390/nu18050856)
Supplement: Supplementary file 1 [file nutrients-18-00856-s001.zip › nutrients-4153233-supplementary.pdf]

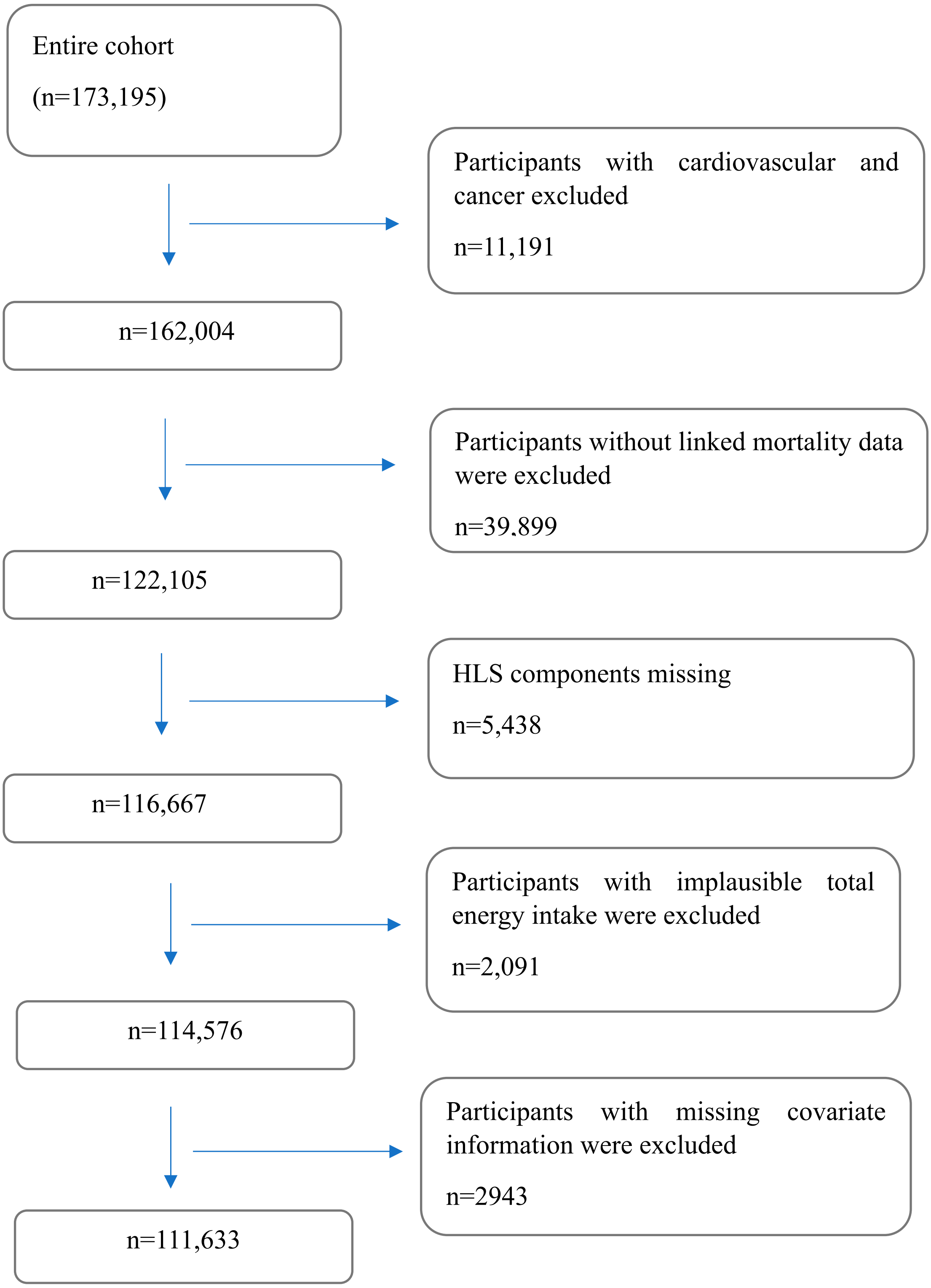

Supplementary figure S1. Flowchart of Inclusion and Exclusion of the Study Population

Supplementary table S1. Baseline characteristics for the overall and included cohorts by sex

Characteristics	Men		Women		Men[a]	Women[b]
	Overall cohort	Included	Overall cohort	Included	P-value	
n	59,258	39,423	113,937	72,210		
Age, years	55.84(9.43)	55.74(9.38)	54.53(8.54)	54.82(8.48)	0.1276	<0.0001
**Smoking status, n (%)**						
Never	16,453(27.71)	10,813(27.42)	108,573(95.29)	69,568(96.34)		
Former	23,716(40.02)	15,705(39.84)	1,489(1.31)	915(1.27)	<0.001	<0.001
Current	18,667(31.50)	12,905(32.73)	2,673(2.35)	1,727(2.39)		
Alcohol consumption, g/day	18.23(38.77)	18.77(38.30)	1.94(8.70)	2.07(9.49)	<0.001	<0.001
BMI, kg/m^2	24.39(2.74)	24.39(2.73)	23.70(2.96)	23.58(2.94)	<0.001	<0.001
History of hypertension, n (%)	13,345(22.52)	8,387(21.27)	20,194(17.72)	12,004(16.62)	<0.001	<0.001
History of hyperlipidemia, n (%)	5,634(9.51)	3,790(9.61)	10,041(8.81)	6,600(9.14)	<0.001	<0.001
Physical activity (<150)	48,721(82.22)	32,234(81.76)	95,699(83.12)	59,537(82.45)	0.686	0.0002
Physical activity (≥150)	10,537(17.78)	7,189(18.24)	19,238(16.88)	12,673(17.55)		
uPDI score	52.77(6.64)	52.20(6.49)	52.68(6.58)	51.94(6.43)	<0.001	<0.001

[a] P-value comparing overall cohort vs. included among men; [b] P-value comparing overall cohort vs. included among women.

Data are expressed as n (%) or mean (standard deviation)

Supplementary table S2. Association between healthy lifestyle scores and the risk of mortality after excluding participants with baseline diabetes

	No. of cases / Total	0-1	2	3	4	5	P for trend	Per 1-point increment
**All-cause mortality**								
**Men**	2,509 / 36,053	ref	0.832 (0.733-0.943)	0.698 (0.615-0.792)	0.606 (0.523-0.702)	0.677 (0.535-0.857)	<0.0001	0.864 (0.830-0.900)
**Women**	1,860 / 68,641	ref	0.593 (0.407-0.864)	0.514 (0.354-0.744)	0.423 (0.290-0.618)	0.397 (0.257-0.612)	<0.0001	0.842 (0.795-0.892)
**Cancer mortality**								
**Men**	1,110 / 36,053	ref	0.792 (0.657-0.954)	0.640 (0.530-0.771)	0.562 (0.452-0.700)	0.636 (0.449-0.902)	<0.0001	0.845 (0.795-0.898)
**Women**	925 / 68,641	ref	0.614 (0.357-1.055)	0.528 (0.309-0.900)	0.451 (0.262-0.776)	0.479 (0.262-0.876)	0.0007	0.869 (0.802-0.942)
**Cardiovascular disease mortality**								
**Men**	377 / 36,053	ref	0.790 (0.572-1.090)	0.686 (0.497-0.945)	0.546 (0.373-0.798)	0.415 (0.203-0.847)	0.0003	0.822 (0.740-0.914)
**Women**	304 / 68,641	ref	0.664 (0.243-1.814)	0.577 (0.213-1.563)	0.487 (0.176-1.346)	0.356 (0.107-1.190)	0.0158	0.837 (0.724-0.967)

Models adjusted for age (years), educational level (<7 years, 7–12 years, >12 years), history of hypertension (yes/no), history of hyperlipidemia (yes/no), family history of cardiovascular disease (yes/no), family history of cancer (yes/no), baseline diabetes (yes/no), and menopausal status (yes/no) and hormone replacement therapy use (yes/no) for women only.

Supplementary table S3. Association between a BMI category-modified healthy lifestyle score* and the risk of all-cause and cause-specific mortality

	No. of cases/Total	HR	No. of cases/Total	HR	P for interaction
All-cause mortality	3,063/39,423		2,183/72,210		
0-1	196	1(ref)	12	1(ref)	
2	761	0.813(0.695-0.952)	168	0.854(0.475-1.535)	
3	1,205	0.571(0.491-0.665)	1,227	0.546(0.309-0.964)	0.5773
4	725	0.514(0.437-0.603)	646	0.453(0.255-0.802)	
5	176	0.471(0.383-0.580)	129	0.414(0.229-0.748)	
P for trend	<0.0001		<0.0001		
1 point increment	0.809(0.778-0.841)		0.800(0.752-0.851)		
Cancer mortality	1,319/39,423		1,043/72,210		
0-1	91	1(ref)	4	1(ref)	
2	334	0.764(0.606-0.963)	75	1.249(0.456-3.415)	
3	506	0.512(0.409-0.641)	563	0.818(0.306-2.189)	0.9054
4	312	0.470(0.370-0.596)	329	0.712(0.265-1.912)	
5	76	0.432(0.317-0.590)	72	0.710(0.259-1.945)	
P for trend	<0.0001		0.0004		
1 point increment	0.793(0.743-0.842)		0.854(0.782-0.932)		
Cardiovascular disease mortality	457/39,423		358/72,210		
0-1	26	1(ref)	2	1(ref)	
2	109	0.884(0.576-1.357)	27	0.696(0.165-2.930)	
3	197	0.697(0.462-1.052)	215	0.489(0.121-1.971)	0.9316
4	99	0.519(0.334-0.805)	98	0.386(0.095-1.570)	
5	26	0.514(0.296-0.895)	16	0.298(0.068-1.299)	
P for trend	<0.0001		0.0007		
1 point increment	0.805(0.728-0.890)		0.764(0.653-0.893)		

Models adjusted for age (years), educational level (<7 years, 7–12 years, >12 years), history of hypertension (yes/no), history of hyperlipidemia (yes/no), family history of cardiovascular disease (yes/no), family history of cancer (yes/no), baseline diabetes (yes/no), and menopausal status (yes/no) and hormone replacement therapy use (yes/no) for women only. * Overweight BMI group (25≤BMI<30) was considered as being healthy and assigned a score of 1.

Supplementary table S4. Association between a diet cutoff-modified healthy lifestyle score (25th percentile of uPDI) and the risk of all-cause and cause-specific mortality

	Men		Women	
	No. of cases/Total	HR	No. of cases/Total	HR
**All-cause mortality**	3,063/39,423		2,183/72,210	
0-1	469	1(ref)	42	1(ref)
2	1,022	0.815(0.730-0.909)	631	0.550(0.402-0.753)
3	1,045	0.707(0.633-0.790)	1,110	0.495(0.363-0.674)
4	443	0.637(0.557-0.728)	345	0.411(0.298-0.568)
5	84	0.638(0.503-0.808)	55	0.427(0.285-0.640)
P for trend		<0.0001		<0.0001
**Cancer mortality**	1,319/39,423		1,043/72,210	
0-1	209	1(ref)	17	1(ref)
2	456	0.813(0.689-0.958)	283	0.662(0.405-1.082)
3	431	0.642(0.543-0.760)	525	0.576(0.355-0.936)
4	185	0.586(0.478-0.718)	186	0.526(0.319-0.866)
5	38	0.629(0.443-0.894)	32	0.565(0.313-1.021)
P for trend		<0.0001		0.0064
**Cardiovascular disease mortality**	457/39,423		358/72,210	
0-1	75	1(ref)	7	1(ref)
2	149	0.736(0.557-0.973)	114	0.523(0.244-1.125)
3	160	0.673(0.509-0.890)	180	0.471(0.221-1.005)
4	66	0.586(0.417-0.824)	50	0.378(0.171-0.838)
5	7	0.336(0.154-0.736)	7	0.379(0.132-1.087)
P for trend		0.0003		0.0204

Models adjusted for age (years), educational level (<7 years, 7–12 years, >12 years), history of hypertension (yes/no), history of hyperlipidemia (yes/no), family history of cardiovascular disease (yes/no), family history of cancer (yes/no), baseline diabetes (yes/no), and menopausal status (yes/no) and hormone replacement therapy use (yes/no) for women only.

Supplementary table S5. Association between weighted healthy lifestyle score (0–20 points) and the risk of all-cause and cause-specific mortality

	Men		Women		
Score	No. of cases/Total	HR	Score	No. of cases/Total	HR
**All-cause mortality**	3,063/39,423			2,183/72,210	
0-4	130	1(ref)	0-6	28	1(ref)
5-8	820	0.814(0.677-0.980)	7-13	1,168	0.509(0.350-0.741)
9-12	1,206	0.638(0.531-0.765)			
13-16	757	0.548(0.454-0.663)	14-20	987	0.393(0.270-0.573)
17-20	150	0.512(0.403-0.650)			
P for trend	<0.0001		<0.0001		
1 point increment	0.948(0.938-0.958)		0.932(0.917-0.946)		
**Cancer mortality**	1,319/39,423			1,043/72,210	
0-4	81	1(ref)	0-6	10	1(ref)
5-8	464	0.910(0.682-1.214)	7-13	516	0.645(0.344-1.206)
9-12	474	0.638(0.480-0.849)			
13-16	254	0.549(0.409-0.738)	14-20	517	0.552(0.295-1.034)
17-20	46	0.500(0.344-0.726)			
P for trend	<0.0001		<0.0001		
1 point increment	0.935(0.920-0.951)		0.952(0.931-0.974)		
**Cardiovascular disease mortality**	457/39,423			358/72,210	
0-4	18	1(ref)	0-6	5	1(ref)
5-8	111	0.810(0.546-1.203)	7-13	221	0.507(0.209-1.233)
9-12	193	0.679(0.459-1.006)			
13-16	113	0.607(0.399-0.924)	14-20	132	0.307(0.125-0.753)
17-20	22	0.326(0.153-0.692)			
P for trend	0.0018		<0.0001		
1 point increment	0.957(0.931-0.984)		0.901(0.867-0.937)		

Models adjusted for age (years), educational level (<7 years, 7–12 years, >12 years), history of hypertension (yes/no), history of hyperlipidemia (yes/no), family history of cardiovascular disease (yes/no), family history of cancer (yes/no), baseline diabetes (yes/no), and menopausal status (yes/no) and hormone replacement therapy use (yes/no) for women only. For women, tertiles of the weighted healthy lifestyle score were used instead of quartiles due to the limited sample size.

Supplementary table S6. 2-year or 4-year lag analysis of the association between healthy lifestyle score and all-cause mortality risk

	Exclusion	No. of cases/Total	0-1	2	3	4	5	P for trend	Per 1-point increment
**All-cause mortality**									
Men	2-year	2,883/39,243	ref	0.809 (0.718–0.911)	0.711 (0.631–0.801)	0.627 (0.547–0.718)	0.640 (0.518–0.791)	<0.0001	0.875 (0.843–0.909)
	4-year	2,633/38,993	ref	0.816 (0.720–0.926)	0.706 (0.623–0.801)	0.659 (0.572–0.760)	0.657 (0.528–0.819)	<0.0001	0.885 (0.851–0.921)
Women	2-year	2,077/72,104	ref	0.526 (0.377–0.735)	0.468 (0.337–0.650)	0.376 (0.268–0.526)	0.373 (0.253–0.548)	<0.0001	0.843 (0.799–0.890)
	4-year	1,926/71,953	ref	0.490 (0.349–0.688)	0.436 (0.312–0.609)	0.355 (0.252–0.500)	0.342 (0.230–0.508)	<0.0001	0.842 (0.796–0.891)
**Cancer mortality**									
Men	2-year	1,247/39,243	ref	0.771 (0.646–0.920)	0.638 (0.534–0.762)	0.567 (0.462–0.697)	0.579 (0.419–0.799)	<0.0001	0.846 (0.799–0.896)
	4-year	1,130/38,993	ref	0.764 (0.635–0.920)	0.606 (0.502–0.730)	0.582 (0.471–0.721)	0.571 (0.407–0.799)	<0.0001	0.847 (0.798–0.899)
Women	2-year	1,001/72,104	ref	0.637 (0.377–1.074)	0.548 (0.327–0.918)	0.476 (0.282–0.804)	0.517 (0.290–0.920)	0.0012	0.881 (0.815–0.951)
	4-year	920/71,953	ref	0.572 (0.339–0.967)	0.492 (0.294–0.825)	0.421 (0.249–0.714)	0.465 (0.260–0.834)	0.0009	0.873 (0.805–0.946)
**Cardiovascular disease mortality**									
Men	2-year	431/39,243	ref	0.771 (0.566–1.051)	0.739 (0.545–1.002)	0.625 (0.440–0.888)	0.361 (0.183–0.721)	0.0012	0.852 (0.773–0.939)
	4-year	389/38,993	ref	0.740 (0.531–1.030)	0.747 (0.541–1.031)	0.647 (0.448–0.935)	0.394 (0.198–0.783)	0.0082	0.871 (0.787–0.965)
Women	2-year	340/72,104	ref	0.508 (0.222–1.162)	0.445 (0.196–1.008)	0.369 (0.159–0.854)	0.250 (0.088–0.706)	0.0033	0.817 (0.714–0.935)
	4-year	320/71,953	ref	0.455 (0.198–1.042)	0.408 (0.180–0.925)	0.349 (0.150–0.809)	0.244 (0.086–0.689)	0.0104	0.834 (0.726–0.958)

Models were adjusted for age (years), educational level (<7 years, 7–12 years, >12 years), history of hypertension (yes/no), history of hyperlipidemia (yes/no), family history of cardiovascular disease (yes/no), family history of cancer (yes/no), baseline diabetes (yes/no), and menopausal status (yes/no) and hormone replacement therapy use (yes/no) for women only.
